# Collective States of α-Sexithiophene Chains Inside Boron Nitride Nanotubes

**DOI:** 10.1021/acs.jpclett.4c02977

**Published:** 2025-02-26

**Authors:** Sabrina Juergensen, Jean-Baptiste Marceau, Chantal Mueller, Eduardo B. Barros, Patryk Kusch, Antonio Setaro, Etienne Gaufrès, Stephanie Reich

**Affiliations:** † Department of Physics, Freie Universität Berlin, Berlin 14195, Germany; ‡ Laboratoire Photonique Numérique et Nanosciences, Institut d’Optique, Université de Bordeaux, Bordeaux 33400, France; ¶ Department of Physics, Federal University of Ceará, Fortaleza, Ceará 60455-900, Brazil; § Department of Physics, Technische Universität Berlin, Berlin 10623, Germany; ∥ Engineering Department, Pegaso University, Naples 80143, Italy

## Abstract

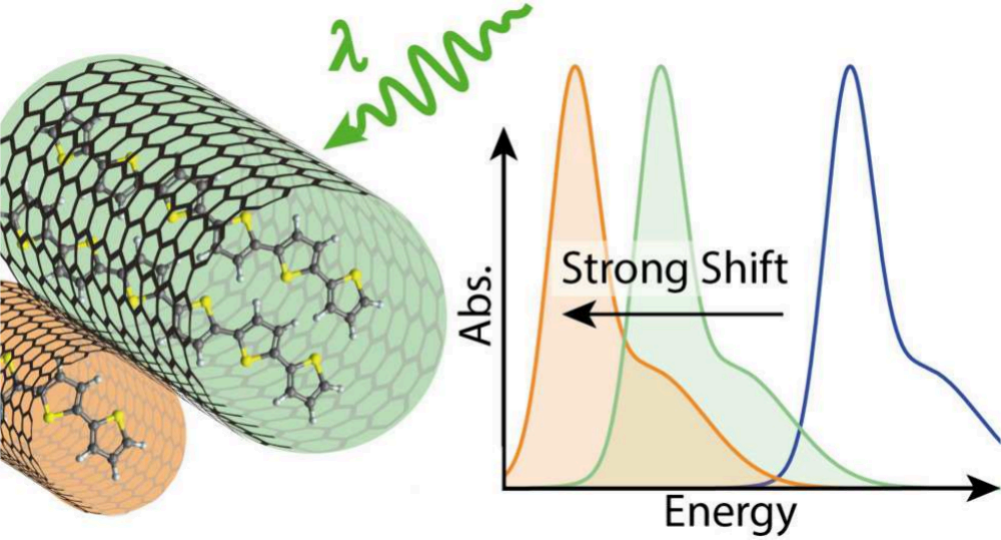

The optical excitation of close-by molecules can couple into collective states giving rise to phenomena such as ultrafast radiative decay and superradiance. Particularly intriguing are one-dimensional molecular chains that form inside nanotube templates, where the tubes align molecules into single- and multifile chains. The resulting collective excitations have strong fluorescence and shifted emission/absorption energies compared to the molecular monomer. We study the optical properties of α-sexithiophene chains inside boron nitride nanotubes by combining fluorescence with far- and near-field absorption spectroscopy. The inner nanotube diameter determines the number of encapsulated molecular chains. A single chain of α-sexithiophene molecules has an optical absorption and emission spectrum that is red-shifted by almost 300 meV compared to the monomer emission, which is much larger than expected from dipole–dipole coupling. For two or more parallel chains, the collective state splits into excitation and emission channels with a Stokes shift of 200 meV due to the chain–chain interaction. Our study emphasizes the formation of a delocalized collective state through Coulomb coupling of the molecular transition moments in one-dimensional molecular lattices. They show a remarkable tunability in the transition energy, which makes encapsulated molecules promising candidates for components in future optoelectronic devices and for analytic spectroscopy.

Collective states in molecular aggregates are fascinating phenomena that indicate their presence by red-shifted, strong, and narrow light emission compared to the single molecule.^1−3^ They are created when the transition dipole moments of the single molecules couple to a macroscopic transition dipole that may exhibit superradiance, with potential applications in nanophotonics and optoelectronics. Collective states emerge in solution when molecules arrange spontaneously in a head-to-tail (J-aggregate) or side-by-side (H-aggregate) configuration. While such states have been studied in molecular aggregates for decades,^4−6^ recent years have shown a growing interest in templating well-ordered molecular lattices to boost size and order of molecular lattices, for example, by growing monolayers of molecules on two-dimensional materials.^2,3,7^ Another fascinating template are nanotubes that may act as perfect containers to align and string molecules into well-ordered one-dimensional chains.^8^ They ensure a close packing of molecules thanks to the nanometer diameter of their inner void.^9−12^ In particular, boron nitride nanotubes (BNNTs) are ideal candidates to observe the strong fluorescence of molecular chains. In contrast to the widely known carbon nanotubes (CNTs),^13^ BNNTs keep the molecular emission intact due to the wide bandgap of boron nitride.^14^ Additional benefits of the nanotube environment are the protection of fluorescent molecules against degradation and photobleaching extending their lifetime and the option to transport and place molecules using the nanotubes as carriers.^13−15^ Nanotubes come with many different structures, electronic, and optical properties allowing for a great variety of molecule-nanotube hybrids.^8^ The inner tube diameter–corresponding to the size of the inner void–was shown to control the number, alignment, and order of molecular chains that form inside a nanotube. For nanotubes <1 nm inner diameter, the template enforces single-molecular chains.^16^ Molecules with a static dipole moment line up in such tubes in a head-to-tail configuration upon filling, which adds up the individual molecular dipoles leading to a macroscopic dipole moment with a strongly nonlinear optical response.^17^ The transient transition moments, on the other hand, couple into collective molecular states.^2,18,19^ This manifests in a giant Raman cross section from molecules aligned in CNTs and an intense red-shifted fluorescence from molecular chains inside BNNTs.^9,13,14^ With increasing inner tube diameter, the molecules form two or more parallel lines or multifile chains, and eventually, unordered molecular aggregates.^9,14,16,20^ The number of parallel chains appears to affect the transition energies of the collective molecular states as identified from a statistical analysis of emission spectra recorded on bulk samples of BNNTs filled with α-sexithiophene (6T) molecules.^16^ However, a detailed understanding of light absorption and emission by single- and multifile molecular chains and their relation to the formation of J- and H-aggregates remains lacking.

Here, we show how the arrangement of 6T molecules in BNNTs determines the properties of their collective molecular states. We observed red-shifted fluorescence and absorption (up to 300 meV shift) from the excitations of single- and multifiled molecular chains compared to the 6T monomers. The overall red-shift of the optical transitions results from the J-type head-to-tail configuration inside the 6T chains. The > 10% red shift of the energy of the collective state compared to the monomer cannot be explained by a point dipole description of molecular transition moments and even appears to exceed a reasonable description with extended transition dipoles. The side-by-side character in multifile chains splits the absorption and emission lines, as is typical in molecular H-aggregates. We complement far-field spectroscopy with wavelength-tunable scattering-type scanning near-field optical microscopy (s-SNOM) to analyze the 6T@BNNT complex with a spatial resolution of 20 nm and resolve absorption along different parts of the BNNT to optically separate the molecular lattices in encapsulation.

Boron nitride nanotubes were obtained from BNNT Materials and the Canadian National Research Center and filled with 6T molecules purchased from Sigmar-Aldrich. The inner tubes diameters have a broad distribution with 70% of the tubes having diameters <3 nm leading to good 6T alignment along the BNNTs axis and the formation of molecular chains.^16^ The encapsulation was carried out under reflux by mixing 6T molecules with freshly opened BNNTs in toluene. The process is well established and described in detail in the Supporting Information. The BNNTs with the encapsulated dye (6T@BNNT) were deposited onto a Si/SiO_2_ wafer by spin coating. The sample was characterized by combined atomic force microscopy (AFM), spatial fluorescence microscopy, and polarization-dependent spatial modulation spectroscopy (SMS).

We distinguished between 6T@BNNT, unfilled BNNT, and free molecular agglomerates by correlating AFM and fluorescence microscopy. A spatial fluorescence map of a 16 × 16 μm sample area was recorded, Figure 1a, using 2.33 eV (532 nm) laser excitation, which is close to the molecular resonance.^13^ It maps the intensity of the 6T fluorescence over the coordinates of the scanned sample area with a resolution of ∼1 μm. The areas of strong fluorescence (red areas in Figure 1a) may originate from filled tubes or free molecular agglomerates on the substrate. To distinguish between agglomerates and filled BNNTs we combine the fluorescence map with an AFM image of the same sample area, see Figure 1b. Thanks for the superior resolution of the AFM (30 nm in this map) we can clearly identify the BNNTs by their elongated shape as opposed to molecular agglomerates or surface residues, but AFM lacks sensitivity toward the presence of 6T. Laying the fluorescence map on top of the AFM image, Figure 1c, shows that the majority of the fluorescent spots indeed come from filled 6T@BNNTs. We identified a strongly emitting 6T@BNNT—likely a bundle of filled tubes—which is marked by a rectangle in Figure 1c. The measurements presented in this paper were performed on this 6T@BNNT structure. Many tubes in Figure 1 were identified as empty, because they did not show up in the fluorescence map despite being present in the AFM image.

**Figure 1:**
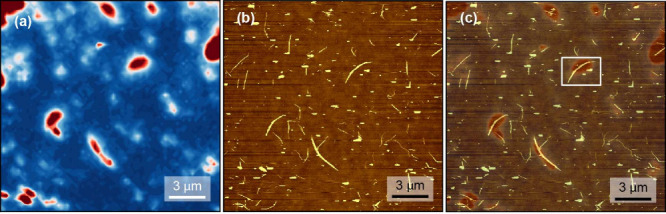
Fluorescence spectroscopy and atomic force microscopy of 6T@BNNT. (a) Spatial fluorescence map of 6T@BNNT on Si/SiO_2_ showing the fluorescence of the main transition (2.10–2.14 eV) of the 6T molecules. Red areas show the highest intensity. (b) AFM topography image of the sample area in panel (a). (c) Fluorescence map on top of the AFM image to identify tubes that are filled with 6T chains. The white rectangle marks the 6T@BNNT bundle that was studied in this work.

**Figure 2:**
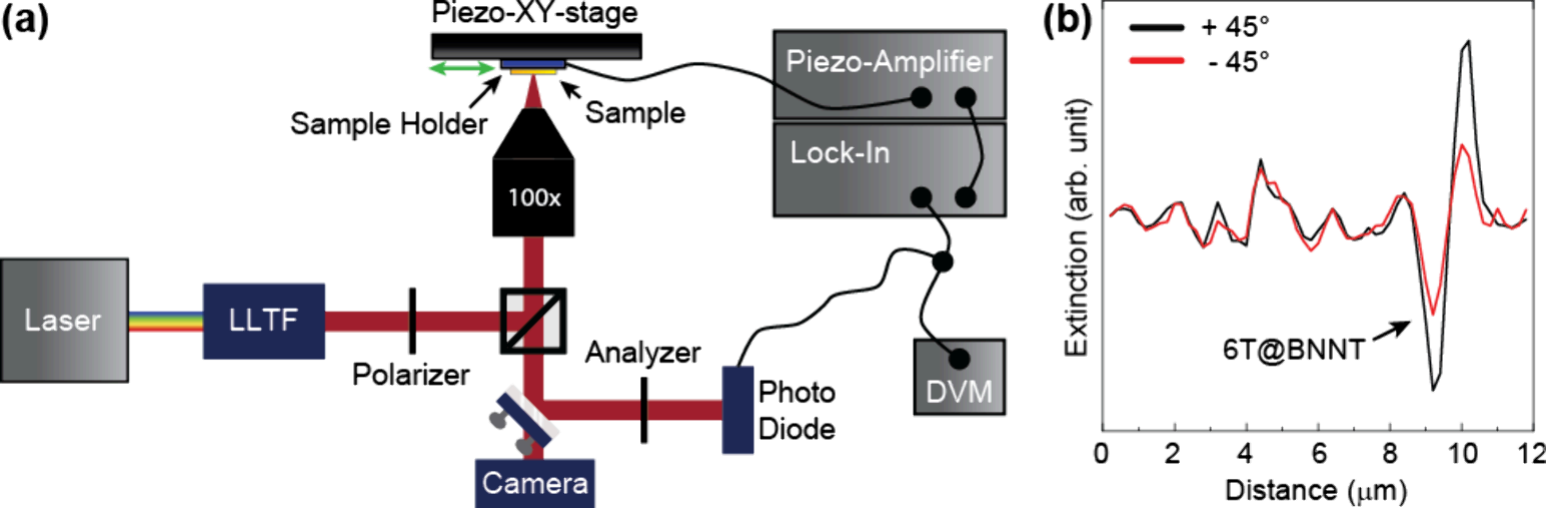
Spatial modulation spectroscopy. (a) Sketch of the spatial modulation setup. (b) Polarization-dependent extinction line profile of a 6T@BNNTs bundle. This measurement was performed on the 6T@BNNTs bundle in Figure 1c using 530 nm excitation wavelength, see Supporting Inforamtion Figure 1 (solid lines) for more information. Black line +45° and red line −45° polarized light.

To analyze the molecular alignment in the filled BNNTs we measured the polarization dependence of the extinction of 6T@BNNT, i.e., the sum of absorption and scattering, using polarized SMS. A sketch of the SMS setup is depicted in Figure 2a, see Supporting Information for details. The sample holder contains a piezo element that modulates the sample position with frequency *f* while the sample is scanned through the focus of the laser beam. The demodulation of the scattered signal with a lock-in amplifier at frequency *f* leads to a contour plot with the shape of the first derivative of a Gaussian profile, from which we determine the extinction cross section of the tube bundle.^21−23^

We first recorded polarization-dependent SMS using 2.34 eV (530 nm) laser excitation, Figure 2b and Supporting Information Figure 1a. When the light is polarized along the 6T@BNNT axis, the extinction is maximal (black line in Figure 2b) and when the polarization is (almost) orthogonal to the tube it is minimal (red line). Other objects like dirt and nonordered molecular agglomerates do not show a polarization dependence, see Supporting Information Figure 1a. This agrees with the previous observations of the polarization dependence of 6T molecules inside BNNTs and CNTs^13,16^ and shows that 6T molecules are aligned along the tube axis. The polarization-dependent SMS measurements also allowed us to identify molecular agglomerates outside BNNTS, see Supporting Information Figure 1a. With the described 6T@BNNT characterization we thus distinguished between 6T@BNNTs, empty BNNTs, 6T agglomerates, and residues outside of BNNTs.

We measured the absorption and fluorescence by the 6T@BNNT bundle marked in Figure 1c to study the optical excitations of the encapsulated 6T chains. Marceau et al.^24^ showed that the fluorescence spectra of BNNTs filled with a varying number of parallel 6T chains differ, presumably due to the interactions between the chains. For our 6T@BNNT bundle with tubes of slightly different diameters we, therefore, expect a superposition of single- and multifile responses in the optical spectra. The fluorescence spectrum is plotted in Figure 3a together with the 6T monomer spectrum measured in solution. In the monomer spectrum, the two main peaks were assigned to the 0–0 (2.44 eV) zero-phonon line and its 0–1 (2.29 eV) phonon replica with an energy separation of 0.15 eV.^25−27^ The emission of the 6T@BNNT is shifted to smaller energies as it is typical for the formation of molecular aggregates.^28,29^ The energetic separation of the two main peaks increased for the encapsulated molecules compared to the free molecules (monomer), Figure 3a and Table 1, and the intensity ratio changed.

**Figure 3:**
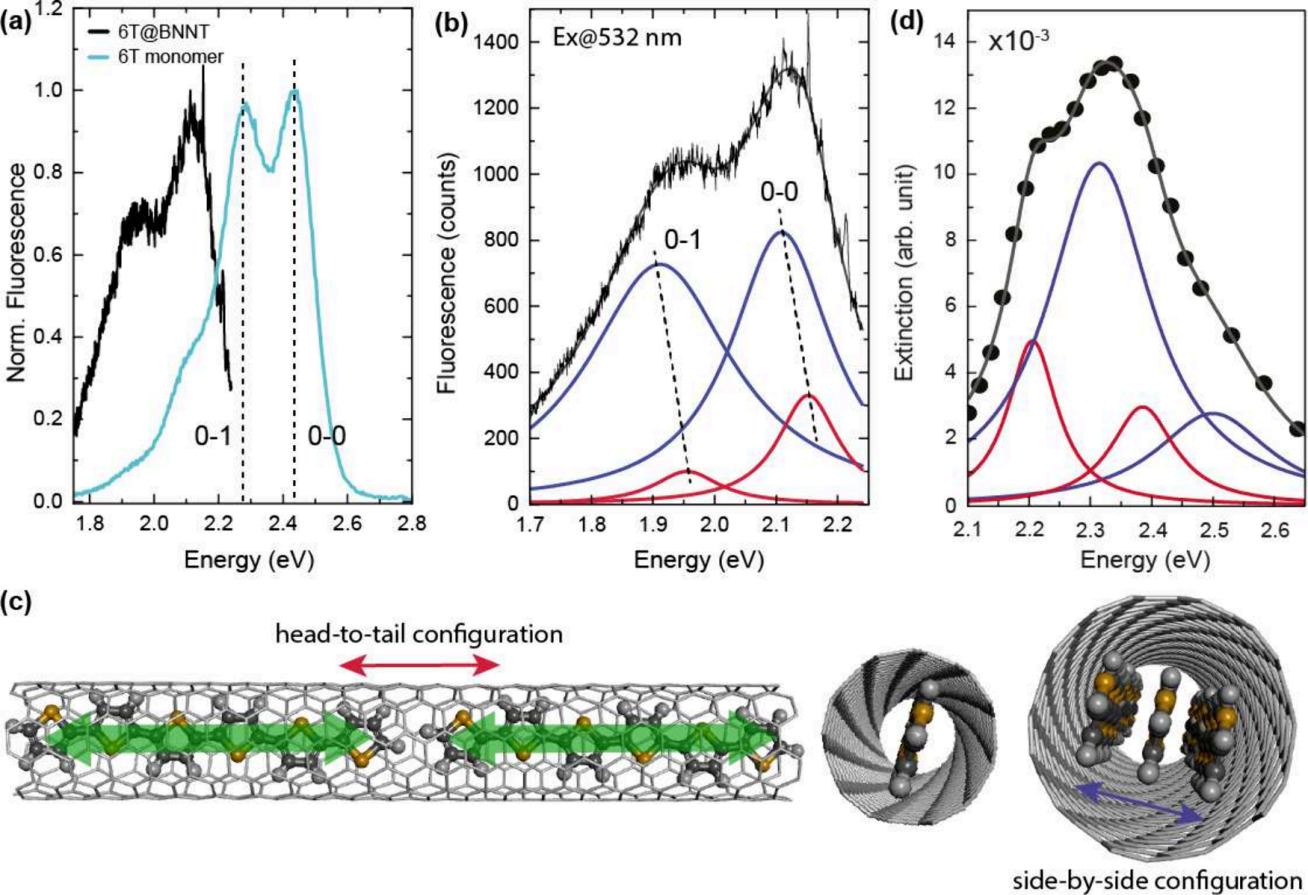
Fluorescence spectra and wavelength-dependent spatial modulation spectroscopy. (a) The fluorescence spectrum of 6T@BNNT (black) and 6T monomer (light blue) show a frequency shift due to the formation of ordered molecular aggregates inside the tube. (b) Fluorescence spectrum and (d) wavelength-dependent extinction profile of the 6T@BNNT bundle (black). Both are fitted by four Lorentzians (red and blue solid lines) representing the single- (red) and multifile (blue) chains. All measurements were recorded on the 6T@BNNT bundle marked in Figure 1c. (c) Scheme of 6T molecules in a BNNT (side and front view). The green arrows indicate the transition dipole moment of the molecules.

**Table 1:** Transition Energies and FWHM of the 6T Monomer and Single- and Multi-file Chains in the 6T@BNNT Bundle^a^

	*S*_0–0_		*S*_0–1_	
	ω (eV)	fwhm (eV)	ω (eV)	fwhm (eV)
**monomer**				
emission	2.44	0.14	2.29	0.15
**single-file**				
emission	2.15	0.12	1.96	0.13
extinction	2.21	0.10	2.39	0.13
absorption	2.16	0.14	2.25–2.40	-
**multifile**				
emission	2.11	0.20	1.91	0.29
extinction	2.31	0.21	2.50	0.23

a The parameters were obtained from the Lorentzian fits; the uncertainty is on the order of 0.02 eV for the transition energies and 0.01 eV for the FWHM.

We used four peaks to fit the luminescence spectrum corresponding to the 0–0 and 0–1 transitions of single- and multifile chains. This implies that our bundle contains tubes of different diameters with varying configurations of the encapsulated molecular chains.^16^ We also considered other possible interpretations for the shifted spectrum such as excimer formation or the assignment of a smaller number of peaks, see note in the Supporting Information, but found these attempts to fail in a consistent interpretation of all optical spectra.^30,31^ The two pairs of peaks (red and blue lines in Figure 3b) obtained from the fits are assign to the zero-phonon line and the phonon replica of the collective molecular states in single- (red) and multifile (blue) chains. Up to three chains may lie next to each other in ordered aggregates in a tube leading to a head-to-tail configuration of the transition dipoles along the tube axis and a side-by-side configuration perpendicular to the axis, Figure 3c.^16,24^ For tubes with an inner diameter of more than 3 nm the molecules start to form unordered aggregates in the tube leading to an optical response further in the near-infrared that was not observed in our spectra.^32^

The Stokes shift is an excellent fingerprint for the type of molecular agglomerate: It becomes vanishingly small for head-to-tail J-aggregates and increases for side-by-side H-aggregate arrangements. To study the absorption of the 6T@BNNT bundle we performed wavelength-dependent SMS, giving us the extinction of the encapsulated chains, Figure 3d, since boron nitride is fully transparent with a constant index of refraction in the studied energy range. The spectrum consists of two main peaks with a splitting Δω = 0.13 eV, smaller than for the 6T@BNNT and monomer fluorescence, Figure 3 and Table 1. The fitting of the extinction curve with four Lorentzians, see Figure 3d, results in two extinction spectra (red and blue) with an 0–0 and 0–1 energy difference of the two optical transitions (∼0.18 eV) that agrees with the differences observed in luminescence and fits to the C=C stretching vibration of sexithiophene (1450 cm^–1^ or 0.179 eV), see Table 1.^15,25,33,34^ Additionally, the fitted extinction peaks are mirror images of their respective 6T@BNNT emission, Figure 3b, as is typical for molecules due to the similar emission/absorption probabilities in the ground S_0_ and first excited electronic state S_1_.^35,36^ Despite their similar appearances, the double peak structure of the emission and extinction spectra have different origin: The two peaks in the extinction spectrum originate from the 0–0 transitions of the single- and multifile chains, while the fluorescence is dominated by the multifile emission and the peaks come from the 0–0 and 0–1 transitions, Figure 3b.

We used s-SNOM to study the 6T@BNNT absorbance and its distribution along the nanotube axis with a resolution of ∼20 nm. s-SNOM determines the local absorbance of nanomaterials from its phase images, see Supporting Information,^37−39^ and allows to study various parts and hotspots along the 6T@BNNT bundle. Using a wavelength tunable laser we measure phase images of the 6T@BNNT bundle as a function of energy. An exemplary phase image recorded at 2.25 eV (550 nm) is shown in Figure 4a. The red areas indicate higher absorption than the white and blue areas. Figure 4b,c show the calibrated phase signal (absorbance) over 1.9–2.7 eV excitation energy at four different positions along the tube, see arrows in Figure 4.

**Figure 4:**
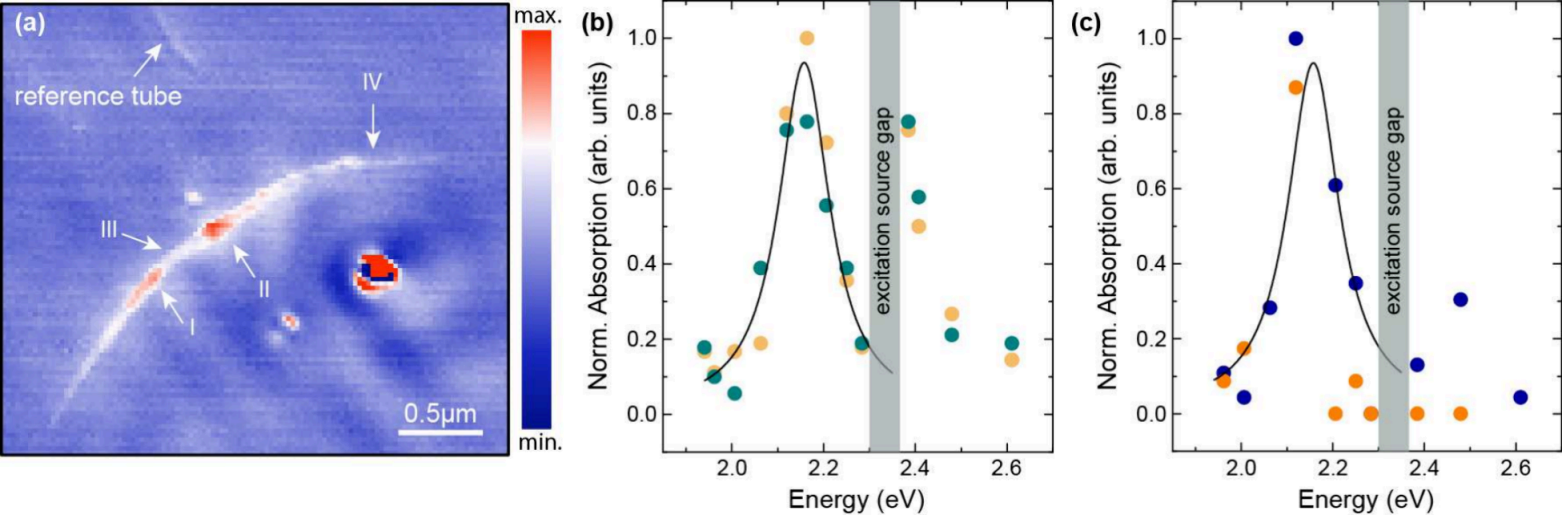
s-SNOM measurements of the 6T@BNNT bundle. (a) s-SNOM phase image of the 6T@BNNT. The red areas indicate stronger absorption than the white and blue areas, see scale. (b) s-SNOM measurements that belong to two parts of the BNNT bundle in (a) with higher absorption, namely position I for yellow and II for petrol symbols. The solid line is a Lorentzian fit of the absorbance. (c) The dark blue (III) and orange (IV) symbols were obtained on to two spots of the 6T@BNNT bundle in (a) with weaker absorption; the black line is a Lorentzian fit. For better comparison the spectra are normalized to unity in panels (b) and (c).

All four positions (I–IV) have an absorption peak at 2.16 eV. Positions I and II appear to show a second absorption maximum between 2.25–2.40 eV, but an energy gap in the tunable laser prevents measurements in this range. We identify the absorption peak at 2.16 eV in Figure 4b,c with the single-file transition at 2.21 eV in the extinction spectrum. The lowest transition of the extinction profile is thus by 50 meV higher than the peak in the absorption spectrum measured with the s-SNOM, see Table 1. This may reflect the characteristic shift between optical near- and far-field measurements; the extinction peaks may also contain a scattering contribution.^40−42^ We also note that the near-field absorption energy is identical to the far-field emission, which would point toward a vanishing Stokes shift for the single file. The variation of the absorbance along the tube bundle indicated that the tubes may be only partially filled or that the tube bundle gets thinner toward its end, since the s-SNOM signal is proportional to the amount of material. This also agrees with the observation that spots III and IV appear to have no absorption associated with the multifile chains.

The fluorescence and absorption spectra of the 6T@BNNT bundle revealed a rich combination of excited collective states, as summarized in the Jablonski diagram in Figure 5, see also Table 1. The diagram compares the energy of the first excited state of the 6T monomer, see Supporting Information Figure 2, with the collective states in 6T@BNNT single- and multifile chains. The red shift of the collective state in the single 6T chain comes from the pure head-to-tail character of the chain. The fluorescence is likewise red-shifted as is typical for J-aggregates.^1,31^ The remaining Stokes shift of 60 meV is vanishingly small compared to the ∼440 meV shift of the monomer, which also indicates a pure head-to-tail configuration.

**Figure 5:**
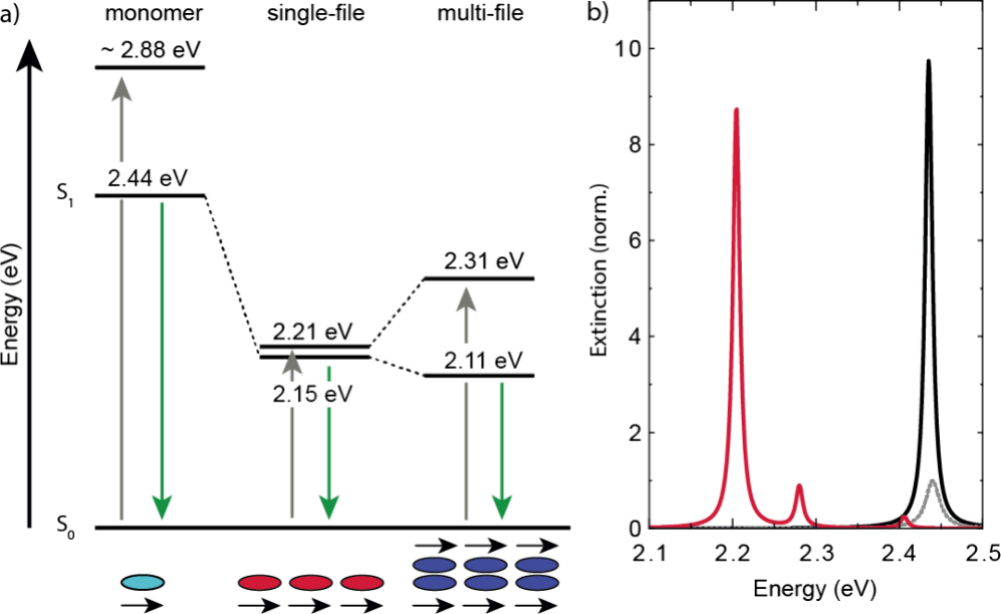
(a) Jablonski diagram of 6T monomer and chains. Absorption (gray arrows) and fluorescence (green) energies. The molecular chains show a red shift of both transitions due to their predominant J-character. The multifile configuration splits the almost degenerate excitation and emission of the single chain, due to the addition of a side-by-side component. The symbols below the diagramm represent a monomer (turquoise), a single- (red) and multifile chain (blue) with their transition moments depicted as black arrows. (b) Calculated extinction of a 6T single-file chain. Calculation in the point (black) and extended dipole model (red); see Supporting Information with *g* = 2.42 nm. Both spectra are normalized to the extinction of a single dipole (gray); ω_0_ = 2.44 eV, *d*_0_ = 8.45 D, and γ_0_ = 5 meV. Further parameters: lattice constant *a* = 2.51 nm, ϵ_*m*_ = 2.55, and number of molecules *N* = 10. The dipole polarization was along the *x* direction.

The collective energies of the single 6T chain in a BNNT agree excellently with previous measurements of optical resonances in 6T@CNTs performed by resonant Raman scattering.^13^ The close similarity of the carbon and boron nitride encapsulated molecules implies that the packing of the 6T is similar in the two types of nanotubes and is governed by the geometry of the available space. The difference in dielectric environment of carbon versus boron nitride nanotube, in contrast, plays a negligible role for the energetic position of the molecular aggregates, which is rather surprising, but agrees well with the identical transition energies of ordered dye monolayers on two-dimensional graphene and hexagonal boron nitride.^2^ It is also consistent with the weak dielectric screening by semiconducting nanotubes measured in double-walled CNTs.^43^

The collective shift of the 6T chains (0.29 eV) is very large compared to the observed red shifts in 2D molecular lattices (0.06 eV).^2,3^ Indeed, simulations of the 6T chains as a string of point dipoles separated by the molecular distance (2.51 nm) predict only a shift of 5 meV, black and dashed gray spectra in Figure 5b, although this same model worked excellently for 2D lattices.^2^ Given the close proximity of the long 6T molecules, the extended size of the transition dipoles needs to be considered,^44^ yielding a good fit to the experimental shifts, red line in Figure 5b. However, the necessary correction for the dipole extension amounts to an effective dipole displacement that is almost as large at the 6T molecules (96.5%), which appears too large. The physical origin of the extremely strong molecule–molecule interaction inside the nanotube remains unclear and should be the subject of further inquiry. One possibility is a waveguiding effect of the encapsulating tube that enhances the Coulomb interaction between the molecular transition dipoles in the direction of the tube axis.

The multifile chain shows a smaller red shift of the absorption and a slightly larger red shift of the fluorescence, i.e., effectively a splitting of the collective single-file state, Figure 5a. The side-by-side configuration of the chains adds an antibonding configuration to the coupling of the transition moments resulting in the splitting. The closer packing of the molecular chains compared to the in-chain separation leads to a stronger splitting of the excited state and thus to an emission that is shifted further to the red compared to the single chain and in a larger Stokes shift (0.2 eV).^31,45,46^ The shift of the absorption peak to smaller energies compared to the 6T monomer confirms that the J-character dominates the optical properties as expected for a multifile molecular chain. Chains with a side-by-side configuration, i.e., in which molecules are oriented perpendicular to the nanotube axis, would result in a collective absorption at energies higher than the monomer and a further increase in the Stokes shift,^1,31^ in contrast to the dual red shift and smaller Stokes shift observed experimentally.

The full width at half-maximum (fwhm) of the 6T@BNNT spectrum further supports our assignment of single- and multifile chains. As a very flexible molecule 6T has a comparatively large fwhm of ∼0.15 eV for the monomer, Table 1. This width was measured in solution, but it remains constant for monomers in a polymethylmethacrylat (PMMA) matrix, Supporting Information Figure 2.^7^ The fwhm of the single chain emission, however, decreases to a mean value of 0.13 eV. The reduction could be due to a reduced motion in the spatial confinement of the BNNT, but this appears unlikely in view of the matrix 6T measurements. We think it reflects the reduced inhomogeneous broadening of the collective state of J-aggregates that comes from the intermolecular coupling and is sometimes called motional narrowing. In line with this, the optical absorption and emission spectra broaden for the multichain file (≳ 0.2 eV). H-aggregates absorb and emit from different vibronic levels of the excited state leading to a broadening of their optical features.^2,47^

Finally, we comment on the relative intensities of the single- and multifile chains in fluorescence, Figure 3. J-aggregates are expected to show brighter emission from their collective state than H aggregates,^2,24,28,48^ but we observed stronger intensity from the multifile chains. This is most likely due to a larger number of tubes with multifile chains in the 6T@BNNT bundle than very narrow tubes with a single file.^16,24^ The emerging H-contribution for the multifile chains is consistent with the relatively low emission intensity of the 0–0 transition in the multichain spectrum compared to 0–1. The 0–0 emission is dipole-forbidden for H-aggregates. Therefore, the peak intensity of the 0–0 emission decreases with increasing number of 6T chains and is expected to vanish for a pure 6T H-aggregate.^7,26,46,49^

In conclusion, we studied absorption and emission from collective optical states of encapsulated 6T chains in BNNTs. Depending on the tubes inner diameter, the 6T molecules arrange in aligned single- and multifile chains inside the nanotubes. The dominant configuration for the energy of the collective state is the J-type head-to-tail arrangement, we did not observe chains where the molecules are aligned in pure H-configuration or randomly orientated inside the tubes. The use of s-SNOM with its high spatial resolution made it possible to resolve which part of the tube bundle has a stronger absorption than other parts which is not possible with standard microscopic methods due to the large laser spot size covering the whole nanostructure. We observed a red-shift of the molecular emission of 0.3 eV in the single 6T chain due to the formation of a collective excited state. Modeling this shift by a chain of extended dipoles required an effective dipole displacement of the entire molecular length, which appears too large for a satisfying explanation. The formation of multifile 6T chains leads to a further red-shift of the molecular emission and an increase of the Stokes shift to 0.2 eV. This agrees with the explanations for an H-type aggregate of two or more long 6T chains. Our study shows the variety of collective states that emerge in molecular chains inside BNNTs. The encapsulation protects molecules against photobleaching and allows to arrange them in artificially large J- and H-aggregates with potential applications in sensing and bioimaging.

## Supporting Information Available

Sample preparation: 6T@BNNT and 6T solution; polarization dependent spatial modulation maps of 6T@BNNT; 6T monomer - optical properties; 6T@BNNT - alternative fit and assignment of the 6T@BNNT bundle emission and extinction; setups: scattering nearfield optical microscopy (SNOM), spatial modulation spectroscopy, atomic force microscpy (AFM), and fluorescence and photoluminescence excitation spectroscopy; and microscopic model of collective dipoles (PDF)
