# Supplementary material for: Collective States of α-Sexithiophene Chains Inside Boron Nitride Nanotubes
Source: J Phys Chem Lett. 2025 Feb 26;16(9):2393–400. doi: 10.1021/acs.jpclett.4c02977 (PMC11891964; doi:10.1021/acs.jpclett.4c02977)
Supplement: Supplementary file 1 — jz4c02977_si_001.pdf [file jz4c02977_si_001.pdf]

Supporting Information: Collective states of α-sexithiophene chains inside boron nitride nanotubes

Sabrina Juergensen,[*,†] Jean-Baptiste Marceau,[‡] Chantal Mueller,[†] Eduardo B. Barros,[¶,§] Patryk Kusch,[†] Antonio Setaro,[†,‖] Etienne Gaufrès,[‡] and Stephanie Reich[†]

†*Department of Physics, Freie Universität Berlin, Berlin, Germany.*

‡*Laboratoire Photonique Numérique et Nanosciences, Institut d'Optique, Université de Bordeaux, Bordeaux, France.*

¶*Department of Physics, Federal University of Ceará, Fortaleza, Ceará, Brazil.*

§*Department of Physics, Technische Universität Berlin, Berlin, Germany.*

‖*Engineering Department, Pegaso University, Naples, Italy.*

E-mail: sabrina.juergensen@fu-berlin.de

Sample Preparation

6T@BNNT: Boron nitride nanotubes (BNNTs) were provided by BNNT Materials and the Canadian National Research Center. α-sexithiophene (6T), toluene, and N,N-dimethylformamide (DMF) were purchased from Sigma-Aldrich and used as received. All used solvents for preparing the samples were of reagent grade quality. For cleaning and opening the nanotubes, the BNNT powder was first annealed for 2 hours at 800°C under atmospheric pressure, followed by sonication in DMF using a cup sonicator until complete dispersion was observed. In the next step, the BNNT solution was centrifuged at 12,000 g; the solution in the upper half of the centrifuge tube was collected and then filtered by a polytetrafluoroethylene (PTFE) membrane with a pore size of 0.22 µm. Encapsulation was carried out under reflux for 48 h at 85°C by mixing 6T molecules and the cleaned and opened BNNTs with toluene in a round bottom flask equipped with a condenser. The concentration of 6T was fixed at 5×10^{-6} M. Intense DMF rinsing of the solution on a PTFE membrane (pore size 0.22 µm) was applied to remove non-encapsulated 6T molecules. Subsequently, soft piranha treatment was performed to remove the last free 6T molecules and the 6T molecules that were adsorbed on the outer wall of the BNNTs.

To deposit 6T@BNNTs via spin coating on a Si/SiO_2 substrate, the 6T@BNNTs were first dispersed in DMF. Before deposition, the Si/SiO_2 substrate was sonicated 10 min in acetone and 10 min in isopropanol for cleaning.

6T solution: The 12 µM solution for the emission and absorption measurements was prepared by dissolving α-sexithiophene purchased by Sigma-Aldrich in toluene ($\geq$ 99.7%) form Honeywell.

Polarisation Dependent Spatial Modulation Maps

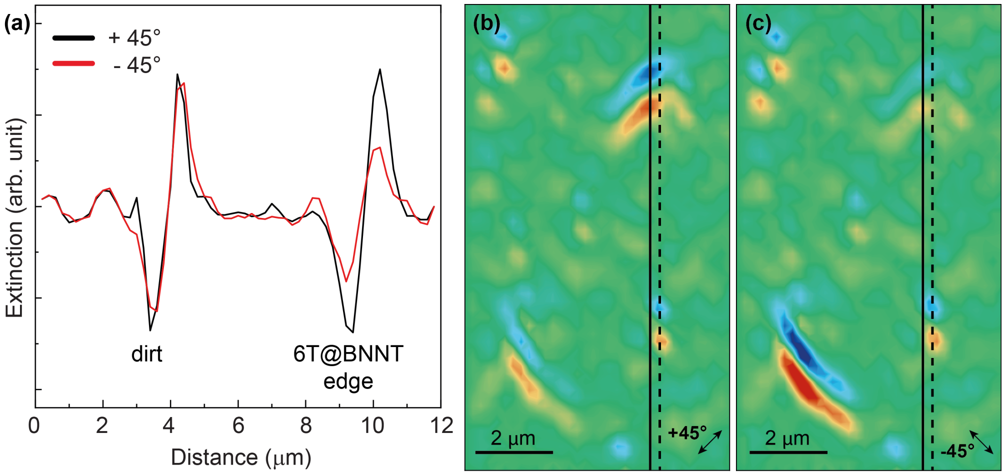

Supplementary Fig. 1: **Polarisation dependent spatial modulation map.** **(a)** Line profile measured by SMS polarizations that are perpendicular to each other, see legend. The intensity of the feature at 4 μm is independent of polarisation and corresponds to dirt or residues on the substrate surface. The 6T@BNNT at 10 μm shows a clear dependence of the optical response on polarisation direction as expected for a one-dimensional structures such as a 6T@BNNT bundle with aligned molecules. **(b,c)** SMS maps of the sample for **(b)** +45° and **(c)** -45° polarised light revealing the pronounced polarisation dependence of the filled 6T@BNNT bundles in the upper right and lower left corners. The solid black lines indicate the line profiles shown in Fig. 2b of the main script. The dashed lines indicate the position of the line profile in **(a)**.

6T Monomer - Optical Properties

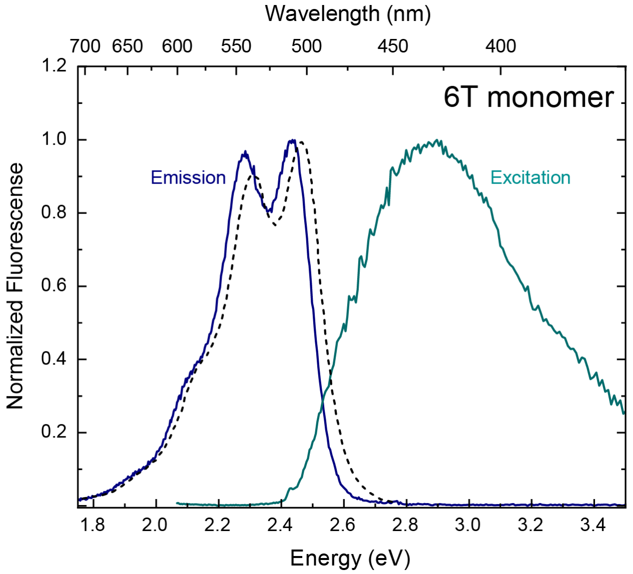

Supplementary Fig. 2: **Emission and luminescence excitation spectrum of 6T monomers.** The dark blue curve shows the emission spectrum of the 6T monomers measured in solution (excited at 2.92 eV, 425 nm). The petrol curve shows the corresponding luminescence excitation spectrum of the 6T solution detected at 2.43 eV (510 nm). The 6T monomers have a Stokes shift of 0.4 eV. The dashed black curve depicts for comparison the photoluminescence of 6T in a PMMA matrix - data taken from Ref. [1]

6T@BNNT - Alternative Fit and Assignment

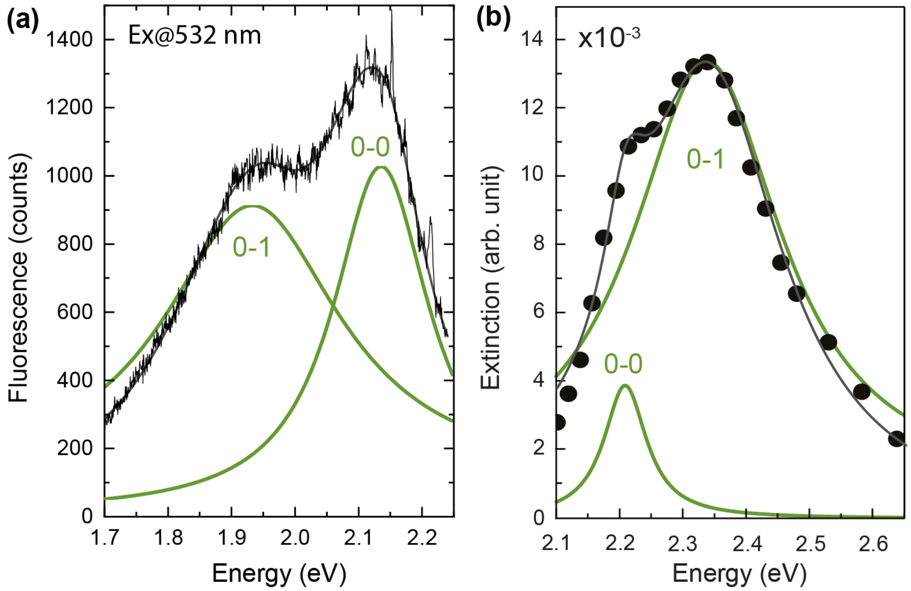

Supplementary Fig. 3: **Fluorescence spectra and wavelength-dependent spatial modulation spectroscopy.** **(a)** Fluorescence spectrum and **(d)** wavelength-dependent extinction profile of the 6T@BNNT (black spheres and lines). Both are fitted by two Lorentzians (green lines) that would correspond to the optical transitions (0-0 and 1-0). The measured data are identical to Fig. 3 of the main manuscript.

We attempted to interpret the luminescence and extinction spectra based on two Lorentzian peaks corresponding to the zero-phonon line and the phonon replica. Supplementary Fig. 3 shows the fit of the 6T@BNNT optical spectra with two Lorentzians instead of the four peaks discussed in the main text. The fits rather poorly reproduce the flanks of the measured spectra. In addition, the extracted peak frequencies, intensities, and widths appear to be inconsistent, see also Supplementary Table 1. For example, the width of the S_{0-0} transition varies by a factor of two between excitation and emission spectrum. Similarly, the splitting of the two transition is 50% larger in the emission than in the excitation channel. We also note the strong decrease in excitation intensity for the 0-0 transition in Supplementary Fig. 3b. This could be explained by a side-by-side orientation (multi-file chain) of the chains, where the 0-0 transition loses intensity or becomes dipole forbidden. However, in this case the line width should increase strongly in contrast to the narrow line width obtained

in the fit. We, therefore, find that the alternative fit did not lead to a consistent model of the optical transition in 6T chains inside BNNTs. Another alternative interpretation would be a contribution by excimer formation in the excitation of the encapsulated 6T. For excimers, the extinction (absorption) spectrum remains unaffected in contrast to the clear change and shift of the absorption spectrum between the monomer, Supplementary Fig. 2, and the 6T@BNNT absorption, Fig. 3.[2]

Table 1: Transition energies and linewidths obtained from the fits in Supplementary Fig. 3.

	S_{0-0}		S_{0-1}	
	ω (eV)	FWHM (eV)	ω (eV)	FWHM (eV)
emission	2.13	0.18	1.93	0.33
extinction	2.21	0.08	2.34	0.29

Setups

Scattering Nearfield Optical Microscope (SNOM)

The s-SNOM measurements were performed with a commercial NeaSNOM from neaspec (attocube) in pseudoheterodyne configuration for background subtraction. As a probe we used platinum coated AFM tips from Nano World (Arrow-NCPt). As an excitation light source a wavelength-tunable laser from Hübner (C-Wave) was used, covering the visible spectral range from 450 to 650 nm.

The laser beam was guided through a beam expander onto a parabolic mirror (NA 0.4) that focuses the beam onto the AFM tip which acts as a near-field probe in the visible spectral range. The incoming laser light creates a strong localized electromagnetic near field at the tip apex. When the tip is in the proximity of the sample, the near field interacts with the sample changing the tip-scattered field. The detected light is composed of near-field signal from sample and background signal.

To extract the near-field signal from the background signal the tip is oscillated at a certain characteristic frequency Ω (300 kHz). For further noise reduction a reference beam is send to a modified Michelson interferometer with oscillating mirror at frequency $M \ll \Omega$. The detected interfering beam is modulated at frequencies $f = n\Omega \pm mM$ leading to side bands next to the fundamental harmonic (nΩ).[3] Here n and m are integers expressing the order of the modulation. Demodulation suppresses the background signal and extracts the phase and the amplitude of the elastically scattered light. The measured phase is proportional to the absorption of the investigated material and the amplitude to its reflectivity. Calibration of the 6T@BNNT phase signal was done by an empty BNNT to account for wavelength-dependent changes in the sensitivity of the setup.

Spatial Modulation Spectroscopy

The extinction was measured with a home build spatial modulation setup. As excitation source an supercontinuum laser (NKT - FIU-15) was used. The laser was coupled to a laser line tunable filter (Photon etc. - LLTF) to filter out single wavelengths. The laser beam was guided to an inverse microscope that was equipped with a xy-piezostage form PI and focused with an 100x objective (NA 0.9) to the sample. The sample was mounted in a special sample holder that was equipped with a piezo-electric element to modulate the sample position at frequency f in the focal spot of the objective. The reflected signal was again collected by the 100x objective and detected with a switchable gain detector (Thorlabs - PDA36A2) that was connected to a lock-in amplifier (AMETEK - Model 7270) to demodulate the signal (ΔR) and further to a digital voltmeter (NI - myDAQ) to measure the total reflection (R). This way the relative change in the reflectance $\Delta R/R$ can be measured which is proportional to the extinction cross section.[4,5]

For the polarisation dependent measurements a polariser was insert into the beam path before the sample and an analyser was added in front of the detector. The analyser was kept constant to avoid changes in the detected signal due to changes in the sensitivity of the setup with polarisation.

Atomic Force Microscpy (AFM)

The AFM images were taken with an XE-150 AFM from ParkSystems in non-contact mode. As a probe a silicon SSS-NCHR AFM tip from Nanosensor was used.

Fluorescence & Photoluminescence Excitation Spectroscopy

The fluorescence measurements of the 6T@BNNT on silicon were performed with a XploRA (Horiba) Raman spectrometer that was equipped with a xy-piezostage from PI. As excitation wavelength a 532 nm green laser diode was used. The spatial fluorescence maps were

taken with an laser power of 0.42 mW and an integration time of 0.5 s. The step size of the piezostage was 250 nm in x and y direction.

The fluorescence of the 6T solution was measured with HORIBA Jobin Yvon Fluorolog-3 spectrofluorometer using a Xe-lamp for illumination. The spectrofluorometer contains a double grating monochromator to select an excitation wavelength and an spectrometer to which an CCD is coupled which can detect wavelengths between 200 nm and 850 nm.

Microscopic Model of Collective Dipoles

To describe the molecule chains theoretically we use the dipole model of Ref.[6] The eletric field influenced by the surrounding dipoles is calculated by the Greens function

$$\begin{aligned} \mathrm{G}(\mathbf{r}_{ij})\mathbf{d}_j =& \frac{k^3}{4\pi\epsilon_0\epsilon_m}\mathrm{e}^{\mathrm{i}kr_{ij}}\left[\left(\frac{1}{kr_{ij}}+\frac{\mathrm{i}}{(kr_{ij})^2}-\frac{1}{(kr_{ij})^3}\right)\mathbf{d}_j\right. \\ & \left. -\left(\frac{1}{kr_{ij}}+\frac{3\mathrm{i}}{(kr_{ij})^2}-\frac{3}{(kr_{ij})^3}\right)(\hat{\mathbf{r}}_{ij}\cdot\mathbf{d}_j)\hat{\mathbf{r}}_{ij}\right], \end{aligned} \tag{1}$$

where $\mathbf{r}_{ij} = \mathbf{r}_i - \mathbf{r}_j$, $r_{ij} = |\mathbf{r}_{ij}|$ represents the distance between the interacting molecules, $\mathbf{d}_j$ the individual dipole moments, and ϵ_m is the dielectric screening by the surroundings. To investigate the influence of the breakdown of the point dipole approximation, we modified the equation by adding a correction that accounts for the finite size of the molecule. In principle, the finite dipole size should affect each of the terms in Eq. 1 differently. However, for simplicity, we chose to simply substitute kr_{ij} in all terms by $k\sqrt{r_{ij}^2 - g^2}$ where g is the same constant for all terms.[7] The dipole-dipole interaction in this system is dominated by the terms proportional to $1/(kr_{ij})^3$, which will be similarly affected by this correction, justifying the use of a single correction parameter. The parameters used for the simulation of the single-file chain are given in the caption of Fig. 5 in the main paper.

References

(1) Loi, M. A.; da Como, E.; Dinelli, F.; Murgia, M.; Zamboni, R.; Biscarini, F.; Muccini, M. Supramolecular organization in ultra-thin films of α-sexithiophene on silicon dioxide. *Nature Materials* **2004**, *4*, 81–85.

(2) Bialas, A. L.; Spano, F. C. A Holstein–Peierls Approach to Excimer Spectra: The Evolution from Vibronically Structured to Unstructured Emission. *The Journal of Physical Chemistry C* **2022**, *126*, 4067–4081.

(3) Ocelic, N.; Huber, A.; Hillenbrand, R. Pseudoheterodyne detection for background-free near-field spectroscopy. *Applied Physics Letters* **2006**, *89*, 101124.

(4) Devadas, M. S.; Li, Z.; Hartland, G. V. Imaging and Analysis of Single Optically Trapped Gold Nanoparticles Using Spatial Modulation Spectroscopy. *The Journal of Physical Chemistry Letters* **2014**, *5*, 2910–2915.

(5) Devadas, M. S.; Devkota, T.; Guha, S.; Shaw, S. K.; Smith, B. D.; Hartland, G. V. Spatial modulation spectroscopy for imaging and quantitative analysis of single dye-doped organic nanoparticles inside cells. *Nanoscale* **2015**, *7*, 9779–9785.

(6) Juergensen, S.; Kessens, M.; Berrezueta-Palacios, C.; Severin, N.; Ifland, S.; Rabe, J. P.; Mueller, N. S.; Reich, S. Collective States in Molecular Monolayers on 2D Materials. *ACS Nano* **2023**, *17*, 17350–17358.

(7) Romaner, L.; Heimel, G.; Ambrosch-Draxl, C.; Zojer, E. The Dielectric Constant of Self-Assembled Monolayers. *Advanced Functional Materials* **2008**, *18*, 3999–4006.
